# Improving the quality of child anthropometry: Manual anthropometry in the Body Imaging for Nutritional Assessment Study (BINA)

**DOI:** 10.1371/journal.pone.0189332

**Published:** 2017-12-14

**Authors:** Joel Conkle, Usha Ramakrishnan, Rafael Flores-Ayala, Parminder S. Suchdev, Reynaldo Martorell

**Affiliations:** 1 Doctoral Program in Nutrition and Health Sciences, Laney Graduate School, Emory University, Atlanta, GA, United States of America; 2 Hubert Department of Global Health, Rollins School of Public Health, Emory University, Atlanta, GA, United States of America; 3 Division of Nutrition, Physical Activity and Obesity; National Center for Chronic Disease Prevention and Health Promotion, U.S. Centers for Disease Control and Prevention, Atlanta, GA, United States of America; 4 Department of Pediatrics, School of Medicine, Emory University. Atlanta, GA, United States of America; National Institutes of Health, UNITED STATES

## Abstract

Anthropometric data collected in clinics and surveys are often inaccurate and unreliable due to measurement error. The Body Imaging for Nutritional Assessment Study (BINA) evaluated the ability of 3D imaging to correctly measure stature, head circumference (HC) and arm circumference (MUAC) for children under five years of age. This paper describes the protocol for and the quality of manual anthropometric measurements in BINA, a study conducted in 2016–17 in Atlanta, USA. Quality was evaluated by examining digit preference, biological plausibility of z-scores, z-score standard deviations, and reliability. We calculated z-scores and analyzed plausibility based on the 2006 WHO Child Growth Standards (CGS). For reliability, we calculated intra- and inter-observer Technical Error of Measurement (TEM) and Intraclass Correlation Coefficient (ICC). We found low digit preference; 99.6% of z-scores were biologically plausible, with z-score standard deviations ranging from 0.92 to 1.07. Total TEM was 0.40 for stature, 0.28 for HC, and 0.25 for MUAC in centimeters. ICC ranged from 0.99 to 1.00. The quality of manual measurements in BINA was high and similar to that of the anthropometric data used to develop the WHO CGS. We attributed high quality to vigorous training, motivated and competent field staff, reduction of non-measurement error through the use of technology, and reduction of measurement error through adequate monitoring and supervision. Our anthropometry measurement protocol, which builds on and improves upon the protocol used for the WHO CGS, can be used to improve anthropometric data quality. The discussion illustrates the need to standardize anthropometric data quality assessment, and we conclude that BINA can provide a valuable evaluation of 3D imaging for child anthropometry because there is comparison to gold-standard, manual measurements.

## Introduction

The Multicenter Growth Reference Study (MGRS) and subsequent development of World Health Organization (WHO) Child Growth Standards (CGS) in 2006 provided a single set of reference measurements for children around the globe [1]. The WHO CGS have been commonly adapted for routine use in low- and middle-income countries as well as in some high-income countries, including the US, for purposes of individual growth monitoring, clinical research, and public health program monitoring [2]. With the new reference came a single measuring protocol that could be adopted in health facilities and surveys everywhere [3]; however, the MGRS protocol requires extensive training and supervision, and repeated measurements [3]. The full MGRS protocol is not routinely followed in health facilities, nor is it used in large-scale surveys that evaluate nutrition such as Demographic and Health Surveys (DHS) and Multiple Indicator Cluster Surveys (MICS).

The non-digital, manual measurements currently in use are susceptible to human error [4], and the use of inadequate measuring protocols can increase measurement error. Poor quality child anthropometry is common in both health facilities and surveys, with skinfold thickness, circumferences and child length the most affected [5–9]. Measurement error can cause misclassification of nutritional status at the individual level and overdispersion at the population level; the latter leads to overestimation of the prevalence of abnormal nutritional status.

There is recognition that the quality of child anthropometry should be assessed before using and disseminating the data. DHS, MICS and Standardized Monitoring and Assessment of Relief and Transitions (SMART) Survey methodologies include assessment of anthropometric data quality. The assessments are all loosely based on recommendations from a WHO expert committee convened in 1995 [10], but there are methodological differences. Recently, there have been calls to improve anthropometry quality through the use of technology, and to revisit the 1995 WHO recommendations to standardize data quality assessment [11].

The data we analyze is part of the Body Imaging for Nutritional Assessment Study (BINA), which compared a 3D imaging system to currently recommended non-digital, manual measurements of stature (length and height), arm circumference (MUAC) and head circumference (HC) in 474 children under five years of age in Atlanta, USA. BINA used the AutoAnthro 3D imaging system, a system comprised of an off-the-shelf, low-cost, handheld scanner (Occipital Inc. Structure Sensor, San Francisco, CA, USA) and custom software for scanning children developed by Body Surface Translations, Inc. (BST) (AutoAnthro, BST, Inc., Atlanta, GA, USA). In order to draw conclusions on the ability of 3D imaging to replicate manual anthropometry, the study needed to collect gold-standard manual anthropometry. A thorough analysis of manual anthropometry quality was particularly important for BINA because, unlike previous research on 3D imaging for anthropometry, the study sample consisted of children under five years of age, an age group that can be difficult to measure with manual methods because of noncooperation. This paper describes the training, standardization and data collection methods for manual anthropometry in BINA; evaluates the quality of BINA manual anthropometry; and provides some recommendations for achieving gold standard manual anthropometry and standardizing data quality assessment to guide clinicians, researchers and public health program managers.

## Materials and methods

Primary caregivers of all children participating in BINA gave written, informed consent and the study was approved by the Emory Institutional Review Board.

### Field staff training and standardization

All five field staff selected for the BINA Study held college degrees, with three of the five holding a master’s degree at the time of the study. In August 2016 field staff completed a three-week training led by trainers from Emory University who had extensive experience with anthropometry in clinic, survey, and research settings; including experience in the study used to develop the 2006 WHO CGS. Training consisted of theoretical and practical sessions on 3D imaging and manual measurements, and field staff were trained to function as both anthropometrists and assistants. Emory University faculty and field staff developed training materials and a study manual.

Training culminated with a three-day standardization test for manual anthropometry at a local daycare center, which consisted of a lead anthropometrist from Emory University and all field staff taking repeated measurements of ten children under five years of age. The lead anthropometrist was considered an expert anthropometrist based on previous experience and a reliability test carried out before BINA. As anthropometrists, field staff were assessed on all measurements except weight, and data was analyzed using ENA Software 2011 [12] and a Microsoft Excel Spreadsheet from the United States Centers for Disease Control and Prevention (US CDC) Micronutrient Survey Toolkit [13]. Passing or failing the standardization test was based on accuracy and reliability results for the main measurements of interest (length and height). We determined accuracy by comparing each anthropometrist to the lead anthropometrist and to the mean of all anthropometrists. For reliability, we computed the Technical Error of Measurement (TEM), which determines if anthropometrists get similar results when carrying out repeated measurements. We also used the US CDC spreadsheets to visually present accuracy and reliability results to the anthropometrists, and to assess whether or not an anthropometrist’s first measurement was systematically lower or higher than the second measurement, also known as the measurement effect [3]. There was no evidence of substantial measurement effect. The accuracy and reliability of the lead anthropometrist and all anthropometrists for length and height were similar to results from the MGRS [3] and were classified as the highest ranking of “good” according to the SMART suggested cut-off point of <0.4 cm for both intra-observer TEM and bias from an expert [14]. Based on results of the standardization test, we retained all anthropometrists for the study.

### Sampling, measurement and data entry procedures

Utilizing convenience sampling we recruited and measured children in facilities, primarily daycare centers, in Atlanta, GA, USA from September 2016 to February 2017. For manual anthropometry we followed measurement techniques used to develop the 2006 WHO Growth Standards [3], including measuring children under two years of age lying down and measuring to the nearest tenth of a kg and cm. We measured weight with digital scales with taring function (Rice Lake Weighing Systems, Inc., Rice Lake, WI), stature with an infant/child/adult wooden board (ShorrBoard, Weigh and Measure, LLC, Maryland USA), and circumferences with synthetic measuring tapes (ShorrTape, Weigh and Measure, LLC, Maryland USA). We routinely checked calibration of scales using known weights and replaced damaged measuring tapes.

During data collection sessions, the five field staff split into two teams of two, with the fifth person acting as a floater. The techniques for scans and manual measurements required an assistant, and teammates alternated as the anthropometrist and assistant. Anthropometrists also took turns as a floater, and the main roles of the floater were to prepare children for measurement and to act as a second assistant for younger children. Anthropometry procedures designed for household surveys often contain a role for the child’s parent. However, since we obtained data primarily in daycare centers where the caregivers were not present, a second assistant was needed to hold and position the child.

After taking time to acclimate the child and establish rapport, and after undressing the child (to their diaper or to skin-tight shorts/leotards provided by the study), the field team started with 3D scans which was then followed by manual measurements. The first anthropometrist completed one session, measuring head circumference, MUAC, and length or height. Instead of one anthropometrist completing two sessions concurrently, the field team reversed roles and a new anthropometrist completed her first session of manual measurements. After both team members completed their first session, the process was repeated for the second session. We staggered manual measurements to reduce the likelihood of anthropometrists remembering their first measurement. To further minimize bias in inter-observer error, anthropometrists entered their own measurement results and did not inform the assistant of the number, which is different from the typical anthropometry procedure of the assistant recording results on behalf of the anthropometrist.

### Quality control and data cleaning

Our custom software for electronic data capture included range checks for non-digital measurements to catch data entry errors. If a measurement was below the 0.01 percentile or above the 99.9 percentile, a pop-up box appeared on the screen and the anthropometrist could either re-enter or accept the value. The software also included automatic triggering of a third measurement for non-digital measurements. The triggers were programmed based on the MGRS standards for the maximum allowable difference (≤0.5 cm for head and arm circumference, and ≤0.7 cm for length) [3], but our study differed from MGRS in that we triggered within observer differences while MGRS triggered between observers. For each child, two anthropometrists entered demographic information, including date of birth, separately. Double data entry allowed us to identify discrepancies caused by data entry error, and we referred to the original consent form to make corrections.

For manual anthropometry, field staff received ongoing monitoring and supervision. This included examining digit preference, the percent of intra-observer measurements exceeding the maximum allowable difference, inter-observer TEM, and estimates of bias by comparing to an expert anthropometrist who measured a subsample of children. In addition to comparing to an expert anthropometrist, field staff also periodically compared with each other. Field staff regularly received data quality reports covering all measures of quality and supervision from expert anthropometrists.

### Quality tests

For digit preference, we examined the proportion of non-digital, manual measurements (height, head circumference and arm circumference) with each of the possible digits (0–9) in the tenths place (mm). We analyzed the first measurement from each anthropometrist. Since each child was measured by two different anthropometrists, we included 948 observations in the analysis. We used the Stata SE 13 (StataCorp, College Station, TX, USA) *digdis* package for analysis. We tested for any digit preference with Pearson’s Chi Squared Test. We calculated the difference between the expected and observed percentages and tested for preference of each individual digit using a two-sided Binomial Test. To determine how close our observed proportions were to a uniform distribution, we summed the difference between expected and observed for positive differences only. The sum gives the percentage of digits that would need to be reclassified (moved from an overrepresented digit to an underrepresented digit) to achieve a uniform distribution, which is similar to the Myer’s Blended Index [7] and the Digit Preference Score [14]. We calculated the Digit Preference Score to allow comparisons with other research.

We used the 2006 WHO Growth Standards to calculate z-scores for weight-for-length/height (WHZ), length/height-for-age (HAZ), weight-for-age (WAZ), head circumference-for-age (HCZ), and arm circumference-for-age (ACZ). We assessed the plausibility of z-scores by determining the proportion of measurements flagged as falling outside of the plausible range as defined in WHO macros (WHZ <-5|>5; HAZ <-6|>6; WAZ <-6|>5; HCZ <-5|>5; ACZ <-5|>5) [15]. We also examined flags used in Demographic and Health Surveys (DHS) for the range of plausible length and height measurements. In the WHO Macro, stature measurements outside of the DHS plausible range (lying down 45–110 cm, standing up 65–120 cm) are not flagged, but WHZ scores cannot be calculated and are automatically set to missing. We calculated z-score standard deviations (SD) and analyzed SD disaggregated by age (under and over two years of age) to determine if the quality of measurements differed by age. We also analyzed z-scores for both the average of all measurements (repeated) and the first measurement of the first anthropometrist (single).

We analyzed both intra- and inter-observer error for height, HC and MUAC using repeated measurements. Intra-observer technical error of measurement (TEM) was calculated with the following formula:

TEMintra=∑i=1N(Mi1−Mi2)22*N,

where N is the number of children and *M*_*i*1_*M*_*i*2_ are the closest repeated manual measures for one child by one observer. For inter-observer TEM we compared the average measurement of one observer to the average measurement of another observer for the same child, changing the numerator in the equation above:

TEMinter=∑i=1N(Mij1+Mij22−Mik1+Mik22)22*N

We calculated Relative TEM, also known as %TEM, by dividing TEM by the mean of all the measurements that went into calculating TEM and multiplying by 100 [16]. Total TEM combines intra and inter-observer reliability in the following formula:

$$TotalTEM=\sqrt{{TEMintra}^{2}+{TEMinter}^{2}}$$

For correlation, we calculated the Intraclass Correlation Coefficient (ICC) using two-way mixed effects [17] and an absolute agreement definition in SPSS 20 (IBM Corp., Armonk, NY, USA).

## Results

Sample characteristics in Table 1 show that children under two years of age were overrepresented, and that there was substantial size, racial, and ethnic variation in the sample.

**Table 1:** Sample characteristics.

Age in months, mean (range)		25.7	(0–59)
Age Groups			
	Newborn (<1 month)	82	(17%)
	1–11.9 months	66	(14%)
	1 year	75	(16%)
	2 years	85	(18%)
	3–4.9 years	166	(35%)
Sex			
	Female	228	(48%)
Race			
	Black	201	(42%)
	White	134	(28%)
	Asian	40	(8%)
	Multiple, Other or Not Reported	99	(21%)
Ethnicity			
	Non-Hispanic	385	(81%)
	Hispanic	77	(16%)
	Not Reported	12	(3%)
Stature in cm, mean (range)		82.3	(42.5–119.0)
Head Circumference in cm, mean (range)		45.7	(31.4–55.3)
Arm Circumference in cm, mean (range)		15.4	(8.8–25.3)

### Quality tests

#### Digit preference

All measurements (stature, HC, and MUAC) showed evidence of terminal digit preference when we tested the child’s first measurement with Pearson’s Chi-Squared Test (p < .01, n = 948). For all measurements, the terminal digit four was significantly overrepresented. In addition, eight was overrepresented for HC, and six was overrepresented for MUAC (Table 2). The sum of the difference between observed and expected percentages for all overrepresented digits indicated that 5.8% of stature measurements and 9.7% of both head and arm circumference measurements would need to be reclassified to achieve a uniform distribution (Table 2). The digit preference score was 5.5 for height, 8.7 for HC, and 8.3 for MUAC.

**Table 2:** Terminal digit preference expressed as percentage of anthropometrists’ first measurement for height, head circumference and arm circumference ending in .0 to .9 compared to the expected 10% among children 0–4.9 years (n = 948 observations from 474 children), BINA 2017.

Terminal Digit (tenths place)	Height			Head Circumference			Arm Circumference		
	% Observed	% Observed—% Expected	P-Value	% Observed	% Observed—% Expected	P-Value	% Observed	% Observed—% Expected	P-Value
0	8.5	-1.5	0.14	5.0	-5.0	0.00	5.9	-4.1	0.00
1	9.2	-0.8	0.45	9.2	-0.8	0.45	10.7	0.7	0.48
2	11.2	1.2	0.23	11.2	1.2	0.23	8.4	-1.6	0.12
3	10.2	0.2	0.79	10.1	0.1	0.87	9.8	-0.2	0.91
4	14.3	4.3	0.00	14.3	4.3	0.00	14.6	4.6	0.00
5	9.7	-0.3	0.83	6.8	-3.2	0.00	6.4	-3.6	0.00
6	9.8	-0.2	0.91	9.8	-0.2	0.91	12.3	2.3	0.02
7	9.8	-0.2	0.91	9.6	-0.4	0.75	11.6	1.6	0.10
8	9.1	-0.9	0.36	13.3	3.3	0.00	9.7	-0.3	0.83
9	8.1	-1.9	0.06	10.8	0.8	0.42	10.5	0.5	0.55

#### Plausibility & reliability

Using means of repeated measures and cutoffs defined by WHO for biologically plausible z-scores one child (0.2%) was flagged, falling above the range for WHZ, and ACZ. Also, the length of one child (0.2%) was lower than the plausible stature range. With single measures, the length of one additional child was lower than the plausible stature range, and no additional children had implausible z-scores.

**Fig 1:**
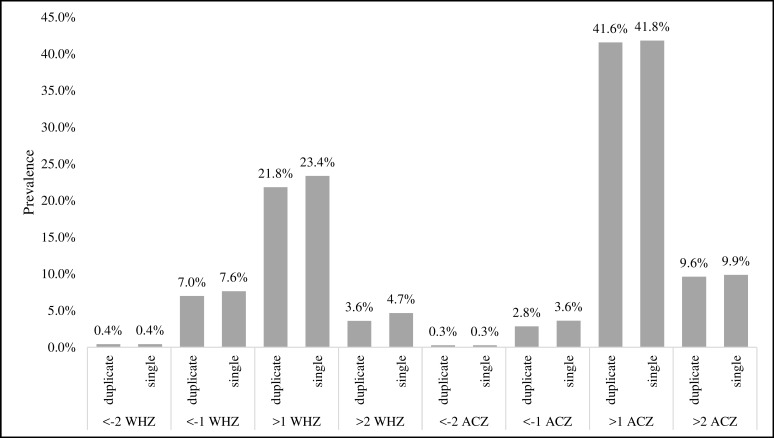
Classification comparison. Prevalence by standard deviation cutoffs for weight-for-height and arm circumference-for-age z-scores for single and means of repeated measures among children 0–4.9 years of age.

For means of repeated measures, the standard deviation for all z-scores (HAZ, WHZ, WAZ, ACZ, and HCZ) was close to 1.0, ranging from 0.92 for WHZ to 1.07 for HAZ. Standard deviations using single measures were slightly higher, with the largest differences in ACZ (0.03) and WHZ (0.02) (Table 3). With increased variation we also found single measures had slightly higher prevalence estimates of children below or above some SD cutoffs for ACZ and WHZ (Fig 1), but differences were not statistically significant when tested with Chi-Square. For both repeated and single measures, z-score standard deviation was not consistently higher for children under 2 years of age compared to children 2 years and older. Levene’s Test for Equality of Variances showed no statistical difference in z-score variance between the age groups for all indices (Table 3), indicating that there was no difference in measurement reliability by age.

**Table 3:** Mean z-score, standard deviation, and test of equal variance between age groups for height-for-age (HAZ), weight-for-height (WHZ), weight-for-age (WAZ), arm circumference-for-age (ACZ), and head circumference-for-age (HCZ) from manual measurements (n = 474), BINA 2017.

		Total (0–4.9 years)			Less than 2 years (U2)			2–4.9 years (O2)			Difference in Variance (Levene's Test)		
		N	Mean z-score	Standard Deviation (SD)	n	Mean z-score	Standard Deviation	n	Mean z-score	Standard Deviation	U2 SD-O2 SD	F	P-Value
Repeated measure mean	Height-for-age	474	-0.29	1.07	223	-0.42	1.10	251	-0.18	1.03	0.08	0.12	0.73
	Weight-for-height	472	0.34	0.92	222	0.32	0.88	250	0.35	0.96	-0.08	0.87	0.35
	Weight-for-age	474	0.06	1.04	223	-0.05	1.02	251	0.17	1.05	-0.04	0.26	0.61
	Arm circumference-for-age	385	0.78	0.94	135	0.84	0.92	250	0.75	0.95	-0.03	<0.01	0.93
	Head circumference-for-age	474	0.24	1.02	223	0.11	1.01	251	0.35	1.01	0.00	0.32	0.57
Single measure	Height-for-age	474	-0.30	1.08	223	-0.43	1.11	251	-0.17	1.02	0.09	<0.01	0.96
	Weight-for-height	471	0.34	0.95	221	0.34	0.92	250	0.34	0.97	-0.05	0.38	0.54
	Weight-for-age	474	0.06	1.04	223	-0.05	1.01	251	0.17	1.05	-0.04	0.31	0.58
	Arm circumference-for-age	385	0.78	0.97	135	0.85	0.98	250	0.75	0.96	0.02	0.25	0.62
	Head circumference-for-age	474	0.24	1.04	223	0.11	1.04	251	0.36	1.03	0.01	0.52	0.47

**Fig 2:**
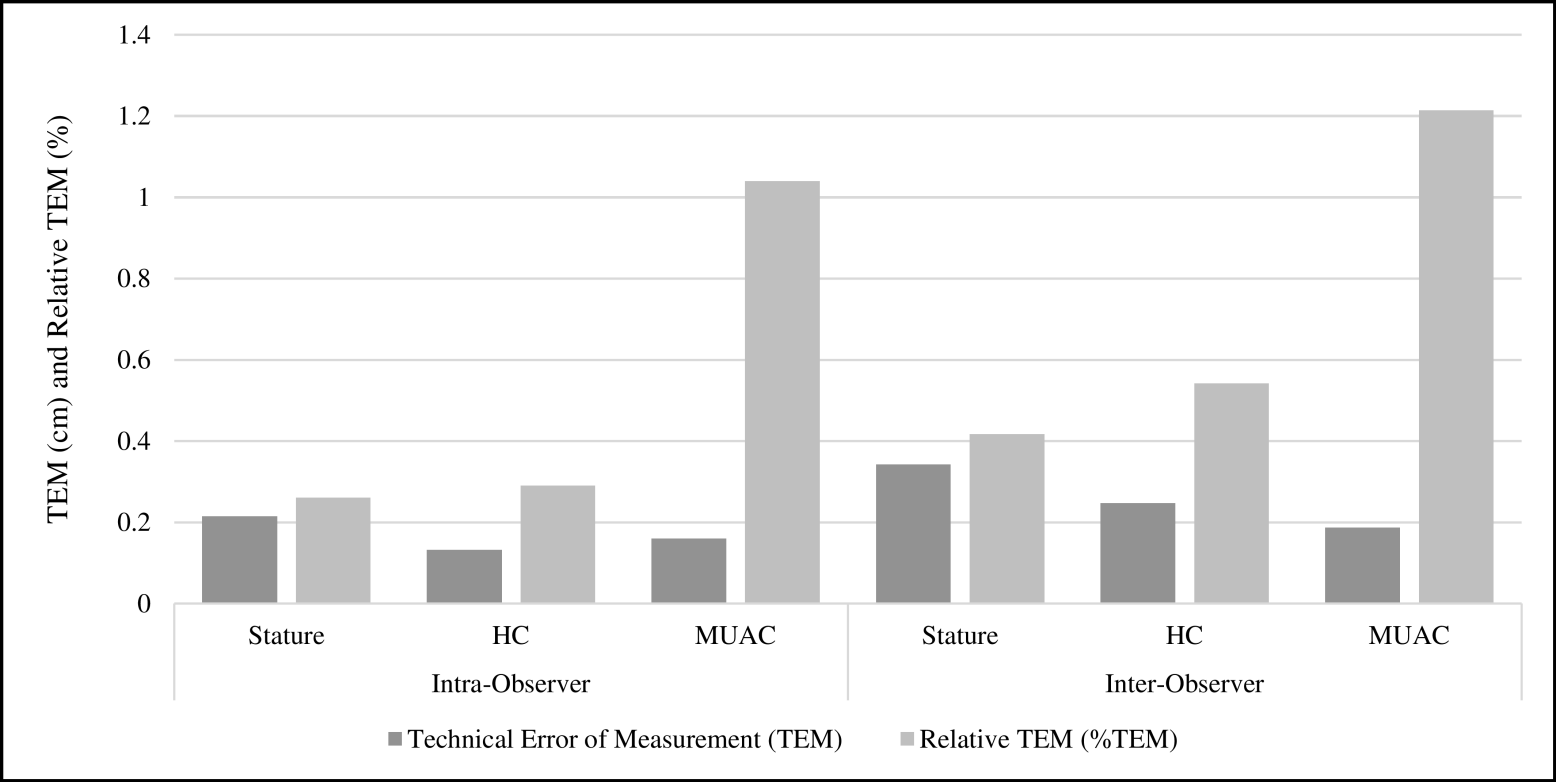
Measurement reliability. Closest two manual measures from single observer (intra-observer) and measurement reliability in average of closest two manual measures between two observers (inter-observer) for stature, head circumference (HC) and arm circumference (MUAC) among children 0–4.9 years of age (intra-observer n = 948, inter-observer n = 474).

The correlation between an anthropometrist’s first and second measurement of a child (intra-observer), and between two anthropometrists’ average measurements of a child (inter-observer) was near perfect. The Intraclass Correlation Coefficient for all intra- and inter-observer measurements was exactly 1.00 (95% confidence interval of 1.00, 1.00) except for inter-observer arm circumference, with ICC of 0.99 (CI: 0.99, 0.99). Intra-observer TEM in centimeters was 0.22, 0.13, and 0.16 for stature, HC, and MUAC respectively. Intra-observer TEM values corresponded to relative TEMs of 0.26%, 0.29%, and 1.04% respectively (Fig 2). As an illustration of TEM interpretation, the stature intra-observer TEM of 0.22 cm means that 2/3^rds^ of repeated measurements were within ±0.22 cm and 95% of replicate measurements were within 2*TEM, or ±0.44 cm. Inter-observer TEM was higher than intra-observer for all measurements: stature TEM 0.34 cm, %TEM 0.42%; HC TEM 0.25 cm, %TEM 0.54%; and MUAC TEM 0.19 cm, %TEM 1.22% (Fig 2). Although MUAC inter and intra TEM was lower in absolute terms, MUAC relative TEM was approximately two to three times higher than stature and HC. Total TEM, combining inter- and intra-observer reliability, was 0.40 for stature, 0.28 for HC, and 0.25 for MUAC in centimeters.

## Discussion

Our data shows that manual anthropometry in BINA was of excellent quality. Only 0.4% of the sample had biologically implausible z-scores or measurements, which is well below the 1.0% cutoff recommended by a WHO expert committee as an indicator of data quality problems [10]. Our z-score SDs also indicated good quality because they were between 0.9 SD—1.1 SD [14, 18], and there were no differences in SD between age groups; a higher SD among children under 2 is an indicator of poor quality and is attributed to difficulty in measuring the length of young, uncooperative children [7, 19]. We found no terminal digit preference for zero or five for stature, and the percent of measurements theoretically requiring reclassification for a normal distribution was low for all measures. We could obtain a normal distribution by changing 6% of terminal digits for our stature measurements compared to an average of 18% in 52 DHS from 2005–2014 [7]. Biological plausibility, z-score SD, and digit preference are metrics commonly used to assess data quality of single anthropometric measures [3, 7–9], and our study showed excellent quality according to all three metrics. In addition, the repeated measures in our study enabled analysis of inter- and intra-observer reliability. For stature the BINA intra- and inter-observer TEMs of 0.22 and 0.34 cm respectively are within the range of inter-observer TEM at MGRS study sites (0.15–0.41 cm) [4]. The TEMs for all of our measurements were on par with TEMs observed at MGRS study sites and were within the 95% precision margin of MGRS expert anthropometrists [4]. The quality of manual anthropometry in BINA was similar to the quality of anthropometric data used to develop the 2006 WHO growth standards.

In our study, we achieved high quality manual anthropometry through following established protocols and adding additional quality control components. We followed advice from MGRS protocol authors to develop a study specific training manual, train and test staff with standardization, and provide adequate supervision during data collection [3]. For measurement techniques, we drew from materials used in household surveys [20–23], which are largely based on a 1986 UN publication on measuring children [24]. We also made sure that our techniques were aligned with the procedures used to develop the WHO Child Growth Standards by referring to MGRS protocol and the WHO anthropometry video used to train MGRS anthropometrists [3, 25]. With the aim to strengthen their understanding of, and dedication to adhere to, study protocols, we included our field staff in development of the study manual. Field staff produced the first draft of the 3D imaging section of the manual and participated in revision and refinement of manual anthropometry sections. Field staff participation in study manual development led to recognition of the need to improve upon some of the borrowed materials. For example, the instructions for finding the mid-upper arm point did not adequately explain identification of the acromion process and illustrations incorrectly depicted measuring the side of the arm. For head circumference, we added additional instructions for the anthropometrists to reposition at both the front and back of the head while removing and replacing the tape multiple times to find the largest circumference. Electronic data capture allowed us to avoid data entry errors through the use of range checks and double data entry at the point of data acquisition; and facilitated adequate monitoring and supervision. Identifying errors during measurement allowed us to re-measure children, reducing the number of implausible measurements. Like the MGRS and following recommendations from a 1995 expert committee [3, 10], we routinely assessed field staff accuracy and provided regular, timely feedback on the quality of anthropometry. Assessment of accuracy during data collection is not standard in common surveys such as DHS and MICS. Repeated measurements, which are rare outside of research study settings allowed us to include reliability in quality monitoring; and electronic data capture provided anthropometrists with real-time feedback on their own reliability through the use of automatic triggers.

There have been suggestions to take repeated measures in surveys [11]. Our results show little difference between single and repeated measures for z-score standard deviations and prevalence. However, with poor quality anthropometry repeated measures should reduce variance. Also, triggering additional measurements provides an incentive to anthropometrists to improve reliability and avoid additional work. For survey institutions with a known history of poor quality anthropometry temporary adoption of duplicate measures by two anthropometrists, along with triggering of repeated measures based on inter-observer maximum allowable difference, may help to improve quality and provide accurate results. Inter-observer error was higher in our study, which is consistent with findings in a developing country setting [19]; the MGRS used inter-observer triggering and adoption of inter-observer triggers in surveys may improve quality more than intra-observer triggers. In addition, repeated measures could be restricted to the most unreliable measures, such as length of children under two years of age and MUAC.

There is some consensus on what to assess for anthropometric data quality. A 1995 WHO expert committee recommended looking at accuracy, reliability, biological plausibility, z-score standard deviation, and digit preference [10]; and these metrics are incorporated into manuals and assessments for DHS and MICS. SMART survey methodology includes all of these metrics, along with additional metrics such as skewness and kurtosis. The 2011 Emergency Nutrition Software (ENA) for SMART has automated reports for standardization tests and data quality, with the latter referred to as a plausibility check [14]. While quality assessments for DHS, MICS and SMART are similar, the methods are not exactly the same. For example, z-score ranges for biological plausibility can differ and there are multiple tests for digit preference. Our study provides a simplified method for assessing digit preference that does not require specialized software. Standardization of what to measure and how to measure anthropometric data quality is needed, but there may be larger institutional differences in how to interpret and act on data quality reports. One of the challenges in this study was determining the criteria to judge whether or not we achieved gold standard manual anthropometry. DHS and MICS do not have indicator thresholds for acceptable anthropometric data quality, and it is very rare for either to suppress data because of poor quality. The ENA SMART plausibility check determines if data quality is acceptable with a composite score based on thresholds for multiple indicators [14]. Another composite scoring system was developed by UNICEF-supported research [9], but the system is not regularly used. For many surveys data quality is assessed, but there is no standard criteria to judge whether or not results should be released.

We can consider z-score standard deviation to illustrate the importance of reaching consensus on interpretation and action. WHO and the US CDC promote the use of normative ranges of SD to determine if survey quality is acceptable [10, 26], but the ranges are based on surveys that have evidence of poor data quality [7, 18]. The most recent DHS data quality assessment showed that 30 of 52 countries had HAZ SD greater than 1.5, but only one country suppressed data because of poor quality [7]. According to SMART data quality is not acceptable if HAZ SD is above 1.2 [14], and a recent modeling study showed that SD of 1.5 can result in substantial overestimation of stunting prevalence [18]. Meanwhile, the published normative range for HAZ SD that some organizations use to deem data quality acceptable is 1.35–1.95 [10, 26]. Digit preference provides another example of the need to focus on interpretation and action. Digit preference is a common phenomenon caused by human tendency to round numbers that has been monitored for years as an indicator of data quality, but even very high measurement digit preference (80% theoretically needing reclassification for a normal distribution) has no meaningful impact on z-score means or prevalences [18]. Weight and height heaping may be useful as a proxy for measuring whether or not anthropometrists follow protocol, but the indicator has little bearing on if and how data should be used. The ENA SMART plausibility check provides a good starting point for consensus. Efforts for standardization should base quality indicator thresholds not only on what is feasible, as was done in the MGRS for reliability and accuracy [3], but also on how quality affects the usefulness of individual growth monitoring, prevalence estimates, and population-level trend analysis.

We designed BINA as a validation study for 3D imaging and did not set out to evaluate how to improve the quality of manual anthropometry. The study design did not allow us to determine the effect of individual protocol components, such as repeated measurements, on data quality. Future studies designed specifically to evaluate quality improvement could help to determine the components of the protocol that are the most cost-effective for quality anthropometry. An additional limitation to our study was that convenience sampling resulted in a sample that did not have normally distributed age, and our findings cannot be extrapolated to a specific age group. Also, while we tried to follow every aspect of the WHO MGRS measuring protocol [3], we did not use the exact same equipment as MGRS; we were unable to procure the metallic measuring tapes used in MGRS and the use of digital length boards was not feasible for our study.

## Conclusions

The high quality manual measurements in BINA can likely be attributed to highly motivated and competent field staff, reduction of non-measurement error through the use of technology, and reduction of measurement error through adequate monitoring and supervision. We made slight improvements to training materials and added to the MGRS protocol for quality control by utilizing electronic data capture and including field staff in the development of the study manual. BINA methodology can be followed to improve anthropometric data quality, but will need to be adapted for use in some contexts. For example, BINA field staff were highly educated and reported child age is accurate in the US; in a different context different strategies may be needed to allow field staff to contribute to manual development, and double data entry may not be sufficient to ensure quality age data. Furthermore, for systematic improvement of anthropometric data in surveys the availability of adequate methodology may not be enough. The lack of consensus on how to interpret and act on analysis of anthropometric data quality leads to survey results being published without reproach, which limits the institutional need and motivation to improve quality. In addition, it may not be feasible to follow the full anthropometry protocol in a clinical setting. Ultimately, while high quality anthropometry is possible with available manual equipment, improved technology may be the most efficient driver of widespread quality improvement; and BINA can provide a good evaluation of 3D imaging technology for child anthropometry because manual measurements were of excellent quality.
